# Ontogeny of a Brazilian Late Triassic Traversodontid (Cynodontia, Cynognathia): Anatomical and Paleoecological Implications

**DOI:** 10.1002/jmor.70047

**Published:** 2025-04-18

**Authors:** Lívia Roese‐Miron, Leonardo Kerber

**Affiliations:** ^1^ Programa de Pós‐Graduação em Biodiversidade Animal Universidade Federal de Santa Maria Santa Maria Rio Grande do Sul Brazil; ^2^ Centro de Apoio à Pesquisa Paleontológica da Quarta Colônia Universidade Federal de Santa Maria (CAPPA/UFSM) São João do Polêsine Rio Grande do Sul Brazil

**Keywords:** Candelária sequence, intraspecific variation, ontogeny, *Siriusgnathus niemeyerorum*, skull anatomy

## Abstract

Investigating the developmental patterns of extinct species provides valuable insights into their anatomy, biology and ecomorphological adaptations. Research on the ontogeny of non‐mammaliaform cynodonts has offered significant contributions to our understanding of these aspects. Here, we aim to describe and discuss the intraspecific and ontogenetic variation of the skull of the Brazilian traversodontid *Siriusgnathus niemeyerorum* (Candelária Sequence, Upper Triassic). We evaluated an ontogenetic series of the species through qualitative comparison and allometric analyses using cranial measures. Our findings reveal several trends during skull growth, including a relative increase in rostrum length, a relative decrease in orbit size, and changes in the zygomatic arch and temporal fenestra proportions. These patterns, when analyzed in the context of the adductor musculature, may be correlated with changes in feeding behaviour, similar to those described for the gomphodontosuchine *Exaeretodon argentinus*. We also report changes in cranial ornamentation, bone fusion, and suture complexity throughout ontogeny. Overall, this study provides a greater understanding of the cranial ontogenetic patterns of *S. niemeyerorum*, contributing to the knowledge of its intraspecific variation. The possible ecological implications of these findings highlight the importance of ontogenetic studies for elucidating the biology of extinct taxa.

## Graphical abstract

In this study, we evaluated an ontogenetic series of the Brazilian traversodontid *Siriusgnathus niemeyerorum* (Candelária Sequence, Upper Triassic), describing the morphological and allometric changes in its skull during the development. Our findings reveal several trends, including a relative increase in rostrum length, a decrease in orbit size, and changes in the zygomatic arch and temporal fenestra proportions. These patterns, when analyzed in the context of the adductor musculature, may suggest a change in feeding behavior during the species' lifetime.

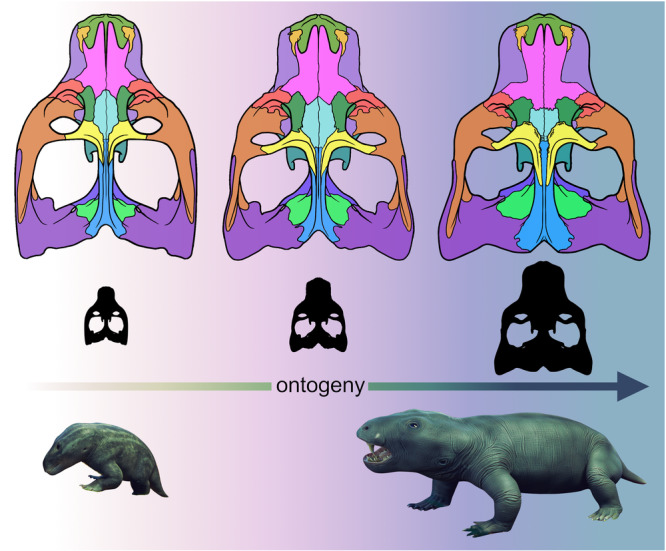

## 1 Introduction

Studying the ontogeny of extinct organisms can inform about not only how their anatomical patterns originate, but also their biology, evolution, and phylogenetic relationships. Direct ontogenetic investigations of non‐mammaliaform cynodonts (i.e., cynodonts that are not Mammaliaformes) have improved taxonomical assignments (Abdala and Giannini 2000; Kammerer et al. 2012), informed about growth patterns (Grine and Hahn 1978; Grine et al. 1978; Bradu and Grime 1979; Botha and Chinsamy 2005; Hopson 2005; Sánchez‐Villagra 2010; Jasinoski and Chinsamy 2012; O'Meara and Asher 2016; Garcia Marsà et al. 2022; Kulik 2023), and raised hypotheses about their feeding behaviour (Grine 2001; Jasinoski et al. 2015; Jasinoski and Abdala 2017; Wynd et al. 2022) and ecology (Botha and Chinsamy 2004).

During the Triassic, a wide diversity of non‐mammaliaform cynodonts occupied various ecological niches in faunas worldwide. Although both of the major subclades of eucynodonts (Cynognathia and Probainognathia, the latter including mammals) likely diverged by the late Early/early Middle Triassic, cynognathians dominated in richness and abundance during their early evolutionary history (Rubidge and Sidor 2001; Abdala and Ribeiro 2010). The Traversodontidae, a particularly diverse group of cynognathians, exhibited a widespread Pangaean distribution (but Gondwanan prevalence; Abdala and Ribeiro 2010; Liu and Abdala 2014; Melo et al. 2015; Abdala and Gaetano 2017; Pavanatto et al. 2018; Abdala et al. 2020; Schmitt et al. 2023; Kerber et al. 2024) from the Middle through Late Triassic (Liu and Abdala 2014; Abdala and Gaetano 2017; Abdala et al. 2020; Hendrickx et al. 2020). These cynodonts are characterized by labiolingually expanded postcanine teeth and dental occlusion, which are traditionally associated with an herbivorous or omnivorous habit (Crompton 1972; Kemp 1980; Hendrickx et al. 2020; Wynd et al. 2022).

In the Upper Triassic strata of southern Brazil (Santa Maria Supersequence, Paraná Basin), traversodontids are some of the most abundant fossils (Schultz et al. 2020). One of these cynodonts is *Siriusgnathus niemeyerorum* (Candelária Sequence), a medium‐sized species of the Gomphodontosuchinae lineage (Pavanatto et al. 2018; Miron et al. 2020). Few comments have been made so far regarding its ontogeny, partially due to the limited number of specimens at the time of description (Pavanatto et al. 2018).

In the last few years, new specimens representing a range of sizes and inferred states of maturity have been collected from sites of the Candelária Sequence, providing a valuable opportunity to investigate ontogeny. In this context, the aim of this study is to analyse an ontogenetic series of *S. niemeyerorum* through qualitative and quantitative methods, to better understand its developmental patterns and provide insights into aspects of its paleoecology.

## 2 Materials and Methods

### 2.1 Specimens

The 13 specimens of *S. niemeyerorum* used in this study were found in Upper Triassic deposits of the Candelária Sequence (sensu Horn et al. 2014) in the municipality of Agudo (Rio Grande do Sul, Brazil), within the Santa Maria Supersequence, Paraná Basin (Zerfass et al. 2003) (see details in Table 1). Some recent biostratigraphical correlations have led to the tentative attribution of the *Siriusgnathus*‐bearing sites to a late Carnian/early Norian age (Miron et al. 2020; Doering et al. 2024; Roese‐Miron et al. 2025). The materials are housed in the Centro de Apoio à Pesquisa Paleontológica da Quarta Colônia, Universidade Federal de Santa Maria (CAPPA/UFSM). They comprise crania with associated lower jaws: CAPPA/UFSM 0032 (holotype), 0109, 0125 and 0260; complete or partial cranial materials: CAPPA/UFSM 0074, 0103, 0124, 0191, 0329, 0330 and 0394; and isolated lower jaws: CAPPA/UFSM 0261, and 0334.

**Table 1:** Provenance of the specimens used in this study. All sites are located in the municipality of Agudo, State of Rio Grande do Sul, Brazil.

Site	Specimens
Niemeyer (29°40′25″ S; 53°14′4.20″ W)	CAPPA/UFSM 0032; 0074; 0103; 0109; 0124; 0125; 0191
ASERMA (29°38′29″ S, 53°16′13″ W)	CAPPA/UFSM 0260; 0329; 0330; 0334
Várzea do Agudo (=Janner) (29°39′10.89″ S, 53°17′34.20″ W)	CAPPA/UFSM 0394
Concórdia (29°38′38″ S, 53°15′39″ W)	CAPPA/UFSM 0261

For comparison, we also analysed first‐hand the South American traversodontids: *Andescynodon mendozensis*—PVL 3833 (holotype), 3834, 3840, 3890, 3892, 3892 a–d, 3893, 3894, 3894‐1, 3899, 3900; *Exaeretodon argentinus* – MACN 18114, 18125, 18200, MLP 43‐VII‐14‐1, 43‐VII‐14‐2 (holotype), 43‐VII‐14‐3, 43‐VII‐14‐4, 43‐VII‐14‐7, 43‐VII‐14‐16, 43‐VII‐14‐17, 61‐VIII‐2‐23, 61‐VIII‐2‐32, 61‐VIII‐2‐37, 61‐VIII‐2‐38, 61‐VIII‐2‐44, PVL 1893, 2056, 2066, 2079, 2082, 2083, 2085, 2093, 2094, 2467, 2468, 2473, 2503, 2564, 2750; *Exaeretodon riograndensis*—CAPPA/UFSM 0033, 0033 A‐E, 0203, 0222, 0227, 0331, 0332, 0395, 0396, MCP PV 1522 (holotype), 2361, 3843, 4278, UFRGS‐PV‐0715‐T, 1095‐T, 1096‐T, 1160‐T, 1161‐T, 1166‐T, 1177‐T; *Luangwa sudamericana*—MCP PV 3167 (holotype), UFRGS‐PV‐0267‐T; *Massetognathus ochagaviae*—MCN PV 2293, MCP PV 3871 (neotype), UFRGS‐PV‐0070‐T, 0125‐T, 0241‐T, 0242‐T, 0243‐T, 0245‐T, 0273‐T, 0712‐T, 1064‐T; *Massetognathus pascuali*—MACN without/number, MCP PV 3284, PULR 10 (holotype), 11, 13, PULR without/number, PVL 3902, 3903, 3904, 3906, 4168, 4439, 4440, 4441, 4442, 4443, 4726, 4727, 4728, 4729, 5441, 5445; *Menadon besairiei*—MCN PV 2750, UFRGS‐PV‐0269‐T, 0434‐T, 0505‐T, 0891‐T, 0903‐T, 0905‐T, 0906‐T, 1054‐T, 1164‐T, 1165‐T; *Paratraversodon franciscaensis*—ULBRA PVT‐049; *Pascualgnathus polanskii*—MLP 65‐VI‐18‐1 (holotype), 65‐VI‐18‐2, PVL 3466, 4416; *Proexaeretodon vincei*—PVL 2565, 3901; *Santacruzodon hopsoni*—MCN PV 2751, 2752, 2768 (holotype), 2770, 2771, MCP PV 4034, 4044, UFRGS‐PV‐0457‐T, 0585‐T, 0586‐T, 1268‐T; *Scalenodon ribeiroae*—UFRGS‐PV‐0239‐T (holotype); and *Traversodon stahleckeri*—UFRGS‐PV‐0224‐T. Comparisons with other taxa were conducted based on the literature.

### 2.2 CT Scanning and 3D Model Generation

To improve the visualization of its basicranial bones, CAPPA/UFSM 0074 was scanned with a SkyScan 1173 X‐ray microtomograph (voltage = 130 kV, current = 61 μA, voxel size = 0.019 mm, number of slices = 2121) at the Instituto do Petróleo e Recursos Naturais (PUCRS, Porto Alegre, Brazil). We manually segmented the specimen in Avizo 3D (FEI Thermo‐Fisher Scientific) and generated 3D surface models of the visible bones.

### 2.3 Anatomical Descriptions and Quantitative Analyses

We briefly characterized each specimen and described the anatomical variation in the sample of *S. niemeyerorum*, focusing on identifying ontogenetic trends in skull shape and anatomy. To determine the growth sequence, we infer relative age (i.e., how young or old an individual is compared to the others) based on size, cranial ornamentation, and suture fusion (see Grine et al. 1978; Abdala and Giannini 2000; Veiga et al. 2018; Wynd et al. 2022). We abstain from attributing specific ontogenetic stages given that this can be misleading in organisms with continuous and/or plastic growth patterns, especially without osteohistological data (Botha and Chinsamy 2001; 2004; 2005; Chinsamy and Abdala 2008; Veiga et al. 2018; Garcia Marsà et al. 2022; Kulik 2023). Thus, individuals are defined as small (presumed juveniles: CAPPA/UFSM 0074, 0103, 0124, 0191, and 0334), medium/intermediate (presumed young adults: CAPPA/UFSM 0125, 0261, and 0329) and large (presumed adults: CAPPA/UFSM 0032, 0109, 0261, and 0330).

**Figure 1:**
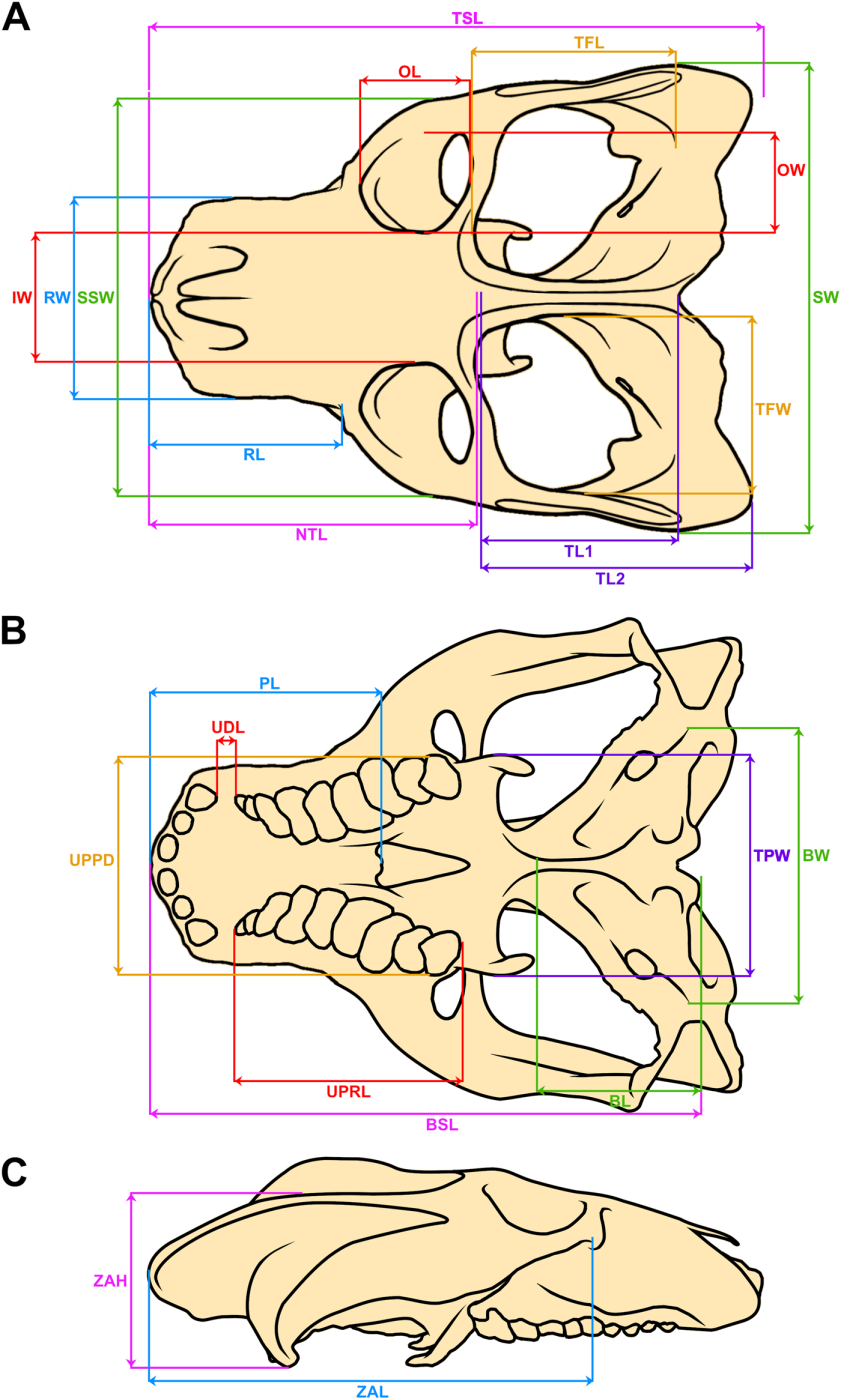
Measurements of the *Siriusgnathus niemeyerorum* specimens. Reconstruction of the cranium in (A) dorsal view; (B) ventral view; and (C) lateral view. BL, Basicranium Length; BSL, Basal Skull Length; BW, Basicranium Width; IW, Interorbital Width; NTL, Non‐temporal Length; OL, Orbit Length; OW, Orbit Width; PL, Palate Length; RL, Rostrum Length; RW, Rostrum Width; SSW, Suborbital Skull Width; SW, Skull Width; TFL, Temporal Fenestra Length; TFW, Temporal Fenestra Width; TL1, Temporal Length 1 (until the posterior limit of the sagittal crest); TL2, Temporal Length 2 (until the posterior limit of the zygomatic arch); TPW, Transverse Process Width; TSL, Total Skull Length; UDL, Upper Diastema Length (between canine and first postcanine); UPPD, Upper Posterior Postcanine Distance; UPRL, Upper Postcanine Row Length; ZAH, Zygomatic Arch Height; ZAL, Zygomatic Arch Length.

To improve our understanding of the allometric patterns throughout growth, we also performed a series of bivariate linear regressions in the statistical software R, v. 4.3.2 (R Core Team 2021). A total of 23 linear measurements were taken from the specimens whenever possible, partially based on Abdala and Giannini (2000) and Wynd et al. (2022) (Figure 1). Four measurements were used separately as the predictor variable in the analyses: two for skull length (basal skull length [premaxilla to exoccipital] and total skull length [premaxilla to squamosal]) and two for skull width (suborbital skull width [width at the level of the orbits] and maximum skull width [width at the widest point of the zygomatic arches]). All other measurements were plotted against these four separately, and the latter were also plotted against themselves. Significance was defined when *p* ≥ 0.05.

### 2.4 Institutional Abbreviations

CAPPA/UFSM, Centro de Apoio à Pesquisa Paleontológica da Quarta Colônia da Universidade Federal de Santa Maria, São João do Polêsine, Brazil; MACN, Museo Argentino de Ciencias Naturales Bernardino Rivadavia, Buenos Aires, Argentina; MCN PV, Museu de Ciências Naturais (Paleovertebrate Collection), Secretaria do Meio Ambiente e Infraestrutura do Estado do Rio Grande do Sul, Porto Alegre, Brazil; MCP PV, Museu de Ciências e Tecnologia da Pontifícia Universidade Católica do Rio Grande do Sul (Paleovertebrate Collection), Porto Alegre, Brazil; MLP, Museo de La Plata, La Plata, Argentina; PULR, Universidad Nacional de La Rioja (Paleovertebrate Collection), La Rioja, Argentina; PVL, Colección de Paleontología de Vertebrados Lillo, Instituto Miguel Lillo, Universidad Nacional de Tucumán, San Miguel de Tucumán, Argentina; UFRGS‐PV‐T, Universidade Federal do Rio Grande do Sul (Paleovertebrate Collection), Porto Alegre, Brazil; ULBRA PVT, former paleontological collection of Universidade Luterana do Brasil, now integrated to the collection of CAPPA/UFSM.

## 3 Results

### 3.1 Characterization of the Specimens

CAPPA/UFSM 0074: Basicranium of a small specimen with preserved stapes. It is broken anteriorly at the cultriform process, and laterally at the lateral margins of the opisthotic.

CAPPA/UFSM 0103: Partial basicranium of a small specimen broken anteriorly at the middle of the cultriform process, with a part of the right squamosal.

CAPPA/UFSM 0191: Cranium of a small specimen slightly dorsoventrally compressed. The specimen is broken anteriorly at the premaxilla. Its surface is badly preserved, and the palatal region is particularly damaged. No teeth are preserved.

CAPPA/UFSM 0124 (paratype): Partial cranium of a small individual, with rostrum, palate, anterior portion of the zygomatic arches, sagittal crest, and part of the basicranium preserved. The canine and incisors are present but broken at their apex. The specimen presents nine alveoli for the upper postcanines (PCs), with the six distalmost PCs preserved on the right side, and the two distalmost on the left side.

CAPPA/UFSM 0329: Complete cranium of medium size in excellent state of preservation, plus some postdentary bones (not figured here). The only teeth preserved in position are the left PC4 and PC5, and both the PC9. This is the best‐preserved cranium of this species, and it is described in Roese‐Miron et al. (in review).

CAPPA/UFSM 0394. Cranium of a medium‐sized specimen without the posterior portion of the zygomatic arches and postorbital bars. The specimen is relatively well‐preserved, with some visible sutures on the skull roof. The basicranium and occipital plate are damaged. It has two incisor alveoli on each side, with only the basal portions of the incisors and the canine preserved, and nine PCs (8 + 1 in eruption).

CAPPA/UFSM 0125 (paratype). Skull (transversely compressed) with two disarticulated mandibular rami. The right zygomatic arch is broken. The surface is badly preserved, especially in the ventral region. It presents two left upper incisors and the basal portion of the right I2, the basal portions of both the canines, and nine PCs (8 + 1 in eruption). The mandibular rami are broken at the symphysis, with no incisor preserved. The coronoid process is partially broken on both sides. There are two right and one left lower incisors preserved, plus the base of the right canine and seven lower postcanines (pcs).

CAPPA/UFSM 0032 (holotype). Complete skull (diagonally distorted but well‐preserved) of a large specimen with disarticulated lower jaws. It presents two upper incisors (lacking the right I2) and a large canine, and it lacks most of the PCs except for the erupting PC9. The mandibular rami are broken at the symphysis; the region of the incisors and canines is mostly absent, with only a part of the right canine alveolus present. There are eight pc alveoli (7 + 1 in eruption), with pc2‐8 present on the right side.

CAPPA/UFSM 0330. Cranium of a large specimen without mandible. The anteriormost portion of the rostrum (premaxilla, left side of the maxilla, and anterior portion of the palate) is broken. The material is relatively well preserved and undeformed. The region of the incisors and canines is completely missing. There are nine PCs (8 + 1 in eruption), with the PC1 and PC2 missing.

CAPPA/UFSM 0260. Fragmented skull (cranium and articulated mandible) of a large specimen transversely compressed. The right side is mostly present, but the left side is completely crushed. Information regarding the basicranium and palate cannot be recuperated.

CAPPA/UFSM 0109 (paratype). Partial skull of a large specimen with mandibular materials (part articulated and part disarticulated). It is likely the largest specimen available, although the complete length of the skull cannot be measured. It possesses the posteriormost region of the cranium and mandible, limited anteriorly by a diagonal fracture. This fracture crosses diagonally, starting on the left side anterior to the orbit and ending on the right side anterior to the postorbital bar. The right postorbital bar and zygomatic arch are severely damaged. The surface is badly preserved, and there is still some matrix inside the temporal fenestrae and orbits, which hinders the visualization of most sutures. Only five left and two right PCs are preserved. Of the lower jaw, a posterior portion of the left mandibular ramus is present and articulated with the cranium, plus an isolated partial right ramus.

CAPPA/UFSM 0334. Well‐preserved lower jaw of a small individual without the postdentary bones. The coronoid and angular processes are preserved on both sizes. There are three pairs of lower incisors, a small canine, and 7 postcanines +1 in eruption partially covered by matrix.

CAPPA/UFSM 0261. Partial lower jaw and cranium (the latter not figured in this study; see Miron et al. 2020) of a small/medium‐sized individual. The right mandibular ramus is broken at the level of the last postcanine. The left ramus of the dentary is almost complete, lacking the dorsalmost end of the coronoid process. The rostral portion of the mandible is severely damaged, hindering the observation of the incisors, canines, and the more mesial postcanines. There are seven (6 + 1 in eruption) preserved pcs on the left side, and five (4 + 1 in eruption) on the right side.

### 3.2 Intraspecific and Ontogenetic Comparisons

#### 3.2.1 Allometric Relationships

**Figure 2:**
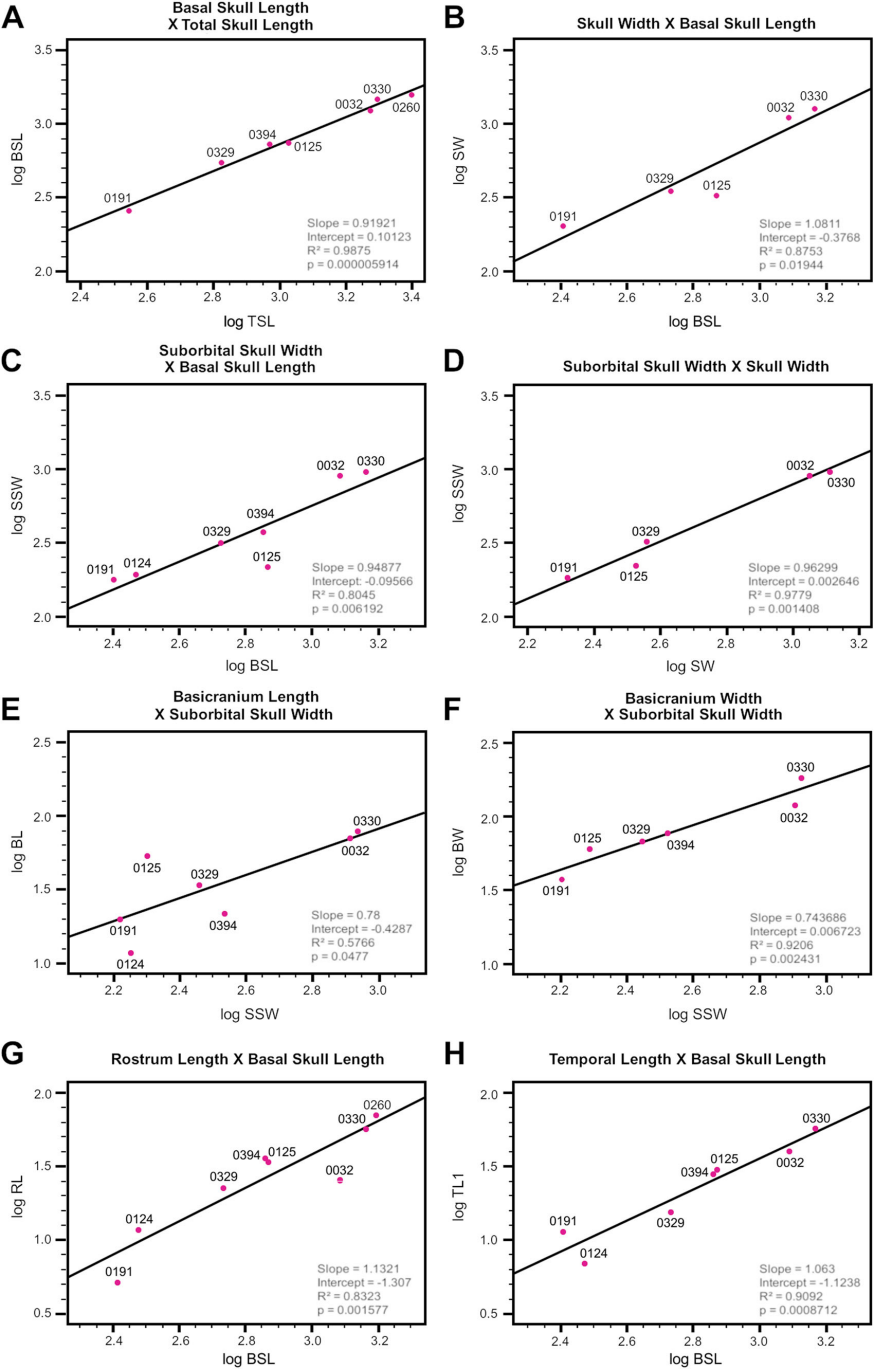
Linear regressions of cranium measurements of *Siriusgnathus niemeyerorum* exhibiting the relationships between: (A) total skull lenght and basal skull length; (B) basal skull length and skull width; (C) basal skull length and suborbital skull width; (D) skull width and suborbital skull width; (E) suborbital skull width and basicranium length; (F) suborbital skull width and basicranium width; (G) basal skull length and rostrum length; and (H) basal skull length and temporal length. The number of specimens included varies (5–8) based on which measurements were possible. All axes are log‐transformed. The statistics (slope, intercept, *R* ^2^ and *p* value) are included in each plot.

All of the measurements (Table 2) except for orbit length (OL), rostrum width (RW), and upper diastema length (UDL) showed a significant (*p *≥ 0.05) allometry with both the basal skull length (BSL) and the total skull length (TSL). In all analyses with the skull length measurements as the predictor variable, the slopes were higher in the analyses that used BSL than the ones that used TSL. This reflects the faster growth rate of the zygomatic arch posteriorly, excluded in the BSL, in relation to the increase of the rest of the skull length (see Figure 2A, which shows that BSL grows slower than TSL). Thus, BSL is likely a more reliable skull length metric than TSL for the analyses performed here, which is also supported by the fact that most of the relationships were statistically stronger in the BSL analyses (lower *p* value and higher *r* ^2^).

**Table 2:** Measurements of the cranium of *Siriusgnathus niemeyerorum* specimens. ‘CAPPA/UFSM’ is occulted from their identification number for brevity.

Measurement (in cm)	0191	0124	0329	0394	0125	0032	0330	0260	0109
BL	3.81	3.03	4.81	3.94	5.85	6.59	6.93	5.59	7.50
BSL	11.11	11.86	15.36	17.39	17.61	21.94	23.71	24.37	—
BW	5.05	—	6.53	6.92	6.16	8.31	9.93	—	11.50
IW	3.16	—	3.26	3.90	3.43	5.025	5.74	—	—
NTL	5.95	—	9.22	11.47	11.11	10.29	13.60	15.81	—
OL	2.28	—	2.94	2.41	3.15	2.45	3.82	3.78	3.3
OW	2.58	—	3.36	3.52	3.21	3.74	4.87	—	5.36
PL	—	5.26	7.11	8.37	7.33	9.47	9.57	—	—
RL	3.39	4.87	6.45	7.94	7.75	6.83	9.67	10.61	—
RW	—	4.32	5.28	6.96	5.01	7.51	—	—	—
SSW	9.58	9.91	12.20	13.17	10.45	19.25	19.75	—	—
SW	10.11	—	12.79	—	12.43	21.03	22.35	—	26.89
TFL	4.28	—	5.88	5.86	7.56	10.47	10.26	—	9.41
TFW	3.91	—	5.04	—	4.70	9.01	8.50	—	9.49
TL1	4.83	3.89	5.48	7.11	7.29	8.28	9.60	—	10.27
TL2	7.13	—	8.71	8.75	11.04	17.07	13.09	12.65	14.43
TPW	—	3.99	4.97	5.83	6.21	6.78	6.92	—	8.25
TSL	12.72	—	16.84	19.44	20.64	26.38	26.96	29.86	—
UDL	—	0.25	0.834	1.50	0.906	1.72	1.69	—	—
UPPD	—	4.48	5.47	6.75	5.91	7.80	7.91	—	8.39
UPRL	—	5.02	6.26	7.42	6.66	7.93	9.36	—	—
ZAH	2.42	—	4.61	—	6.88	7.76	6.62	9.75	—
ZAL	9.71	—	11.83	—	13.66	20.61	17.02	17.51	—

Abbreviations: BL, Basicranium Length; BSL, Basal Skull Length; BW, Basicranium Width; IW, Interorbital Width; NTL, Non‐temporal Length; OL, Orbit Length; OW, Orbit Width; PL, Palate Length; RL, Rostrum Length; RW, Rostrum Width (at the canines level) SSW, Suborbital Skull Width; SW, Skull Width; TFL, Temporal Fenestra Length; TFW, Temporal Fenestra Width; TL1, Temporal Length 1 (until the posterior limit of the sagittal crest); TL2, Temporal Length 2 (until the posterior limit of the zygomatic arch); TPW, Transverse Process Width; TSL, Total Skull Length; UDL, Upper Diastema Length (between canine and first postcanine); UPPD, Upper Posterior Postcanine Distance; UPRL, Upper Postcanine Row Length; ZAH, Zygomatic Arch Height; ZAL, Zygomatic Arch Length.

**Figure 3:**
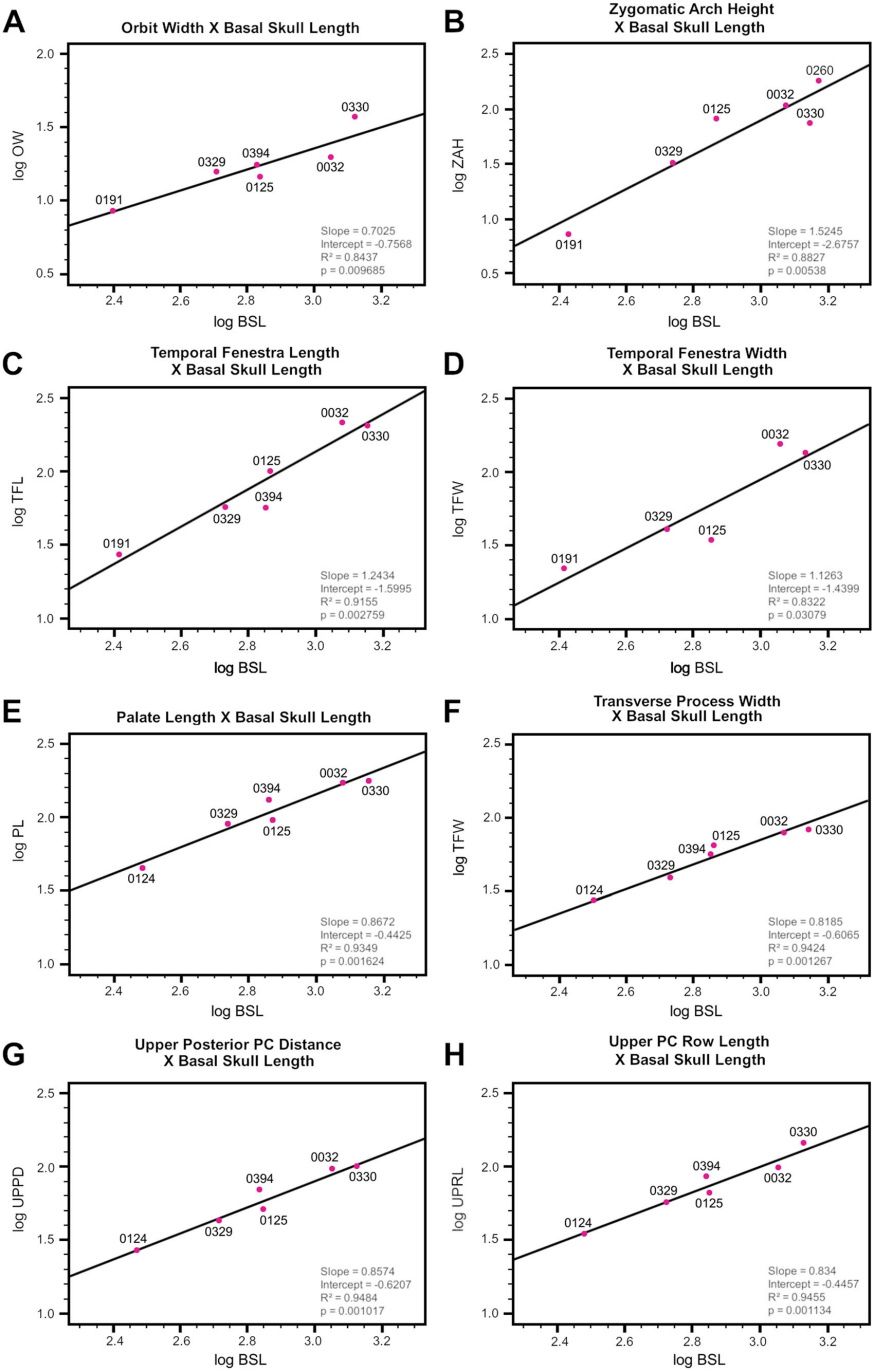
Linear regressions of cranium measurements of *Siriusgnathus niemeyerorum* exhibiting the relationships between: (A) basal skull length and orbit width; (B) basal skull length and zygomatic arch height; (C) basal skull length and temporal fenestra length; (D) basal skull length and temporal fenestra width; (E) basal skull length and palate length; (F) basal skull length and transverse process width; (G) basal skull length and upper posterior postcanine distance; and (H) basal skull length and upper postcanine row length. The number of specimens included varies (5–8) based on which measurements were possible. All axes are log‐transformed. The statistics (slope, intercept, *R* ^2^ and *p* value) are included in each plot.

Most of the measurements increase at a slower rate (slope ≤ 1.0) than skull length. These include many of the width measurements—basicranium width (BW), interorbital width (IW), orbit width (OW, Figure 3A), transverse process width (TPW, Figure 3F), upper posterior postcanine distance (UPPD, Figure 3G)— and some length measurements—basicranium length (BL), palate length (PL, Figure 3E), upper postcanine row length (UPRL, Figure 3H) and zygomatic arch length (ZAL). Contrastingly, the temporal length measurements (TL1 and TL2) approach an isometric pattern (i.e., slope closer to 1.0) (Figure 2H).

The skull width proxies grow approximately isometrically with skull length. The suborbital skull width (SSW) grows slightly slower than skull length (Figure 2C), while the skull width (SW) exhibits the opposite pattern (Figure 2B). This pattern highlights that the width of the skull increases more at the zygomatic arches than at the orbital region. The only measurements that grow significantly faster than the skull length are the rostrum length (RL, Figure 2G), temporal fenestra length (TFL, Figure 3C), temporal fenestra width (TFW, Figure 3D) and zygomatic arch height (ZAH, Figure 3B).

The following measurements showed a significant positive allometric relationship with both of the skull width measurements (SW and SSW): BSL, TSL, RW, PL, UPPD, IW, OW, ZAL, TL1, TL2, TFL, TFW, BL, and BW. All of these measurements appear to increase at a slower rate than the skull width (slope ≤ 1.0), albeit to variable extents (see Figure 2E,F for examples). This pattern is more evident in the SW analyses, a result of the faster increase of this measure in relation to the suborbital skull width. The analyses that more closely resemble an isometric pattern are the skull width measurements (mostly SSW) versus the temporal fenestra measurements (i.e., TFW and TFL), and the skull width measurements plotted against each other (Figure 2D).

#### 3.2.2 Qualitative Comparisons

**Figure 4:**
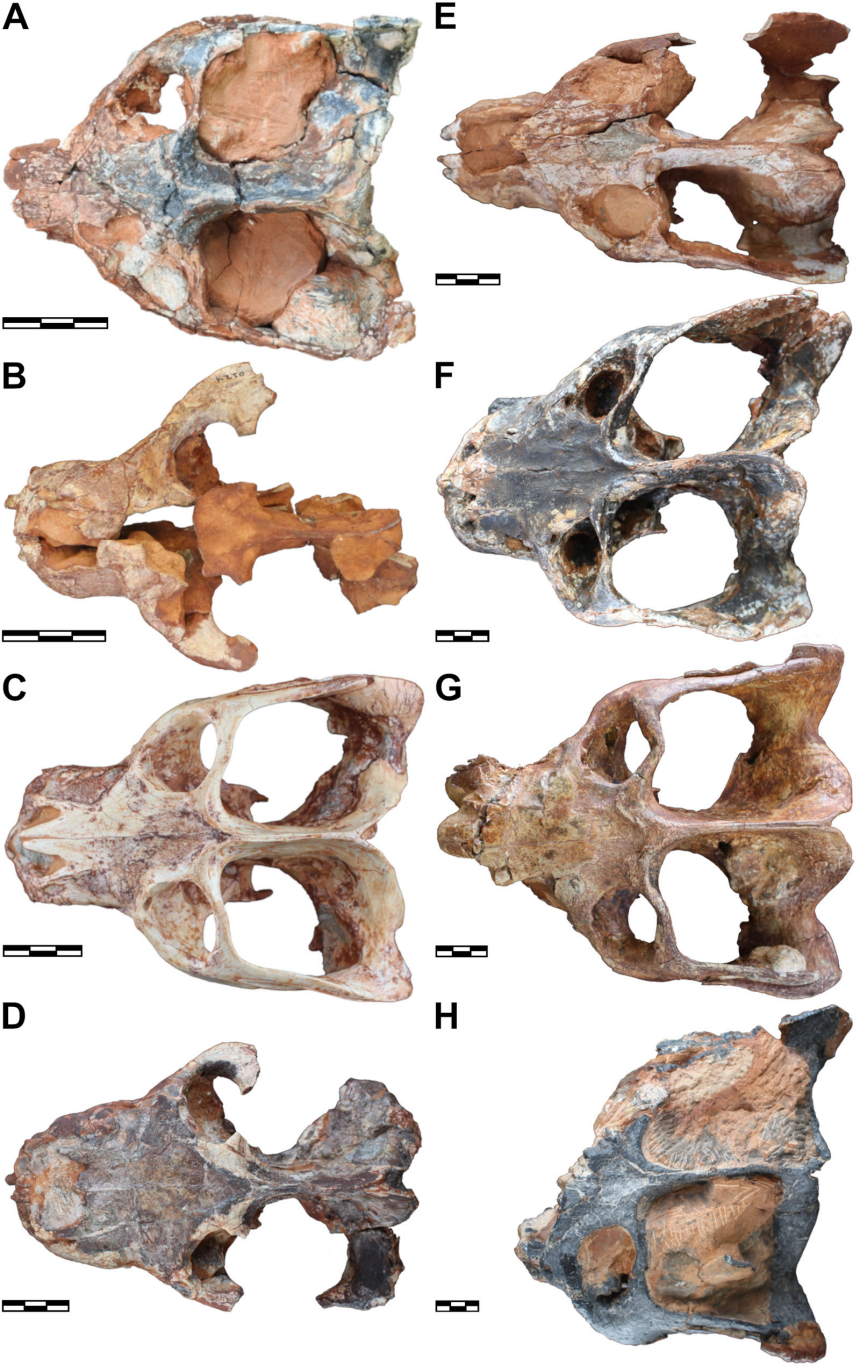
Crania of *Siriusgnathus niemeyerorum* in dorsal view, in ascending order of size. (A) CAPPA/UFSM 0191; (B) CAPPA/UFSM 0124; (C) CAPPA/UFSM 0329; (D) CAPPA/UFSM 0394; (E) CAPPA/UFSM 0125; (F) CAPPA/UFSM 0032; (G) CAPPA/UFSM 0330; (H) CAPPA/UFSM 0109. Scale bars = 30 mm.

**Figure 5:**
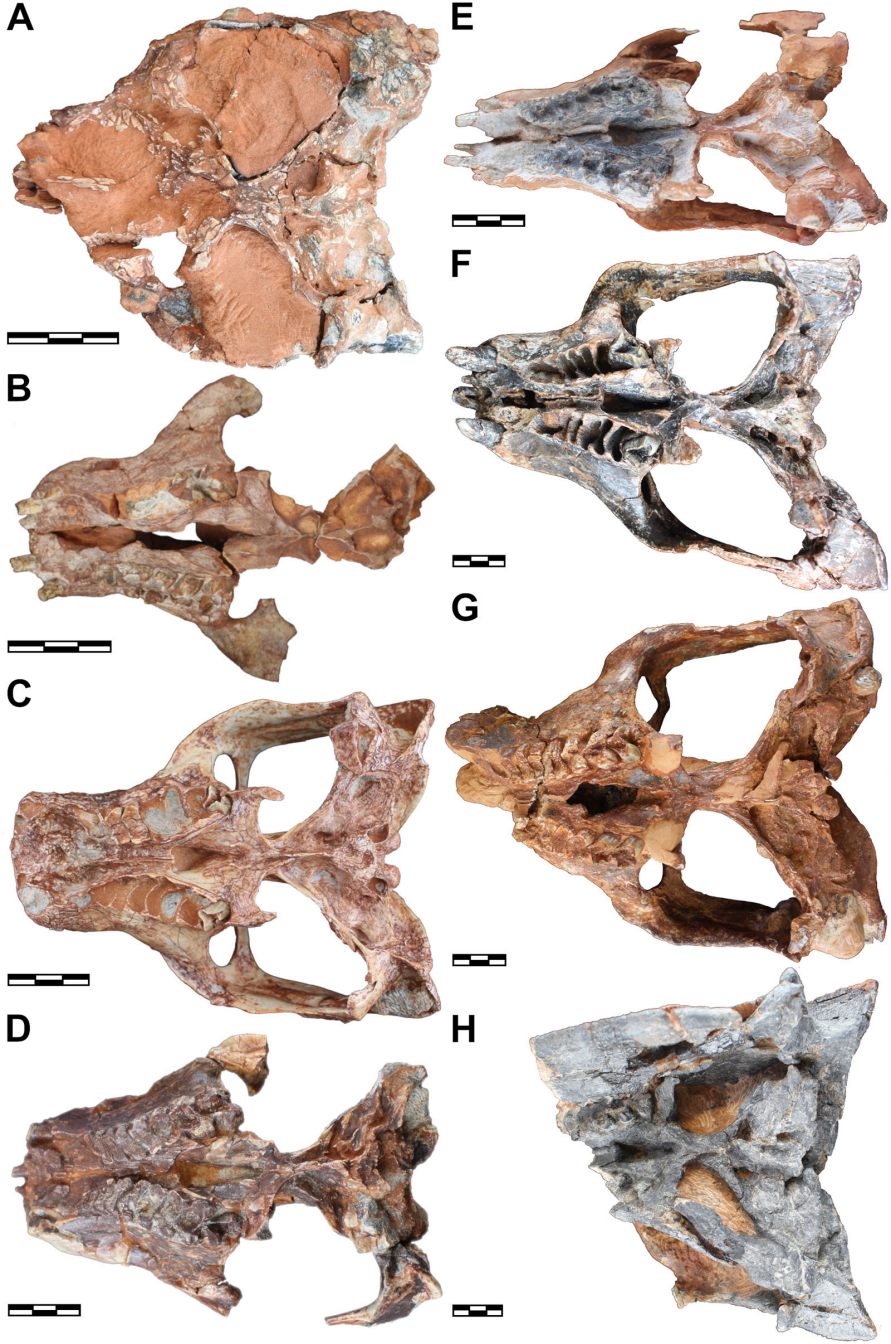
Crania of *Siriusgnathus niemeyerorum* in ventral view, in ascending order of size. (A) CAPPA/UFSM 0191; (B) CAPPA/UFSM 0124; (C) CAPPA/UFSM 0329; (D) CAPPA/UFSM 0394; (E) CAPPA/UFSM 0125; (F) CAPPA/UFSM 0032; (G) CAPPA/UFSM 0330; (H) CAPPA/UFSM 0109. Scale bars = 30 mm.

**Figure 6:**
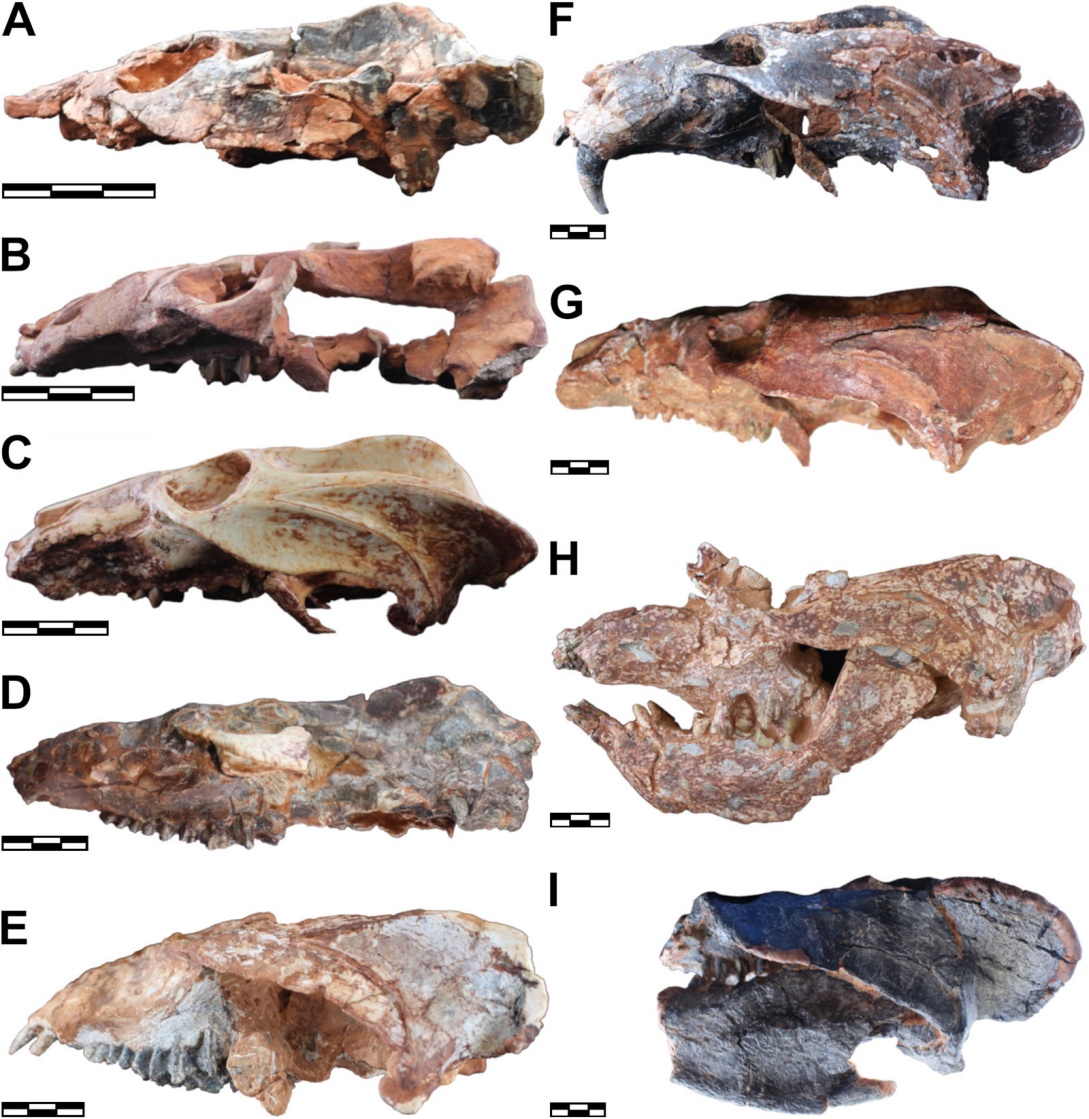
Crania of *Siriusgnathus niemeyerorum* in lateral view, in ascending order of size. (A) CAPPA/UFSM 0191 (lateral right, mirrored); (B) CAPPA/UFSM 0124 (lateral left); (C) CAPPA/UFSM 0329 (lateral left); (D) CAPPA/UFSM 0394 (lateral right, mirrored); (E) CAPPA/UFSM 0125 (lateral left); (F) CAPPA/UFSM 0032 (lateral right, mirrored); (G) CAPPA/UFSM 0330 (lateral right, mirrored); (H) CAPPA/UFSM 0260 (lateral right, mirrored); (I) CAPPA/UFSM 0109 (lateral left). Scale bars = 30 mm.

The rostrum becomes longer and wider during the ontogeny of *S. niemeyerorum* (Figures 4, 5 and 5). In CAPPA/UFSM 0124 (i.e., the smaller specimen with a preserved snout), the rostrum is dorsoventrally short, becoming slightly taller in CAPPA/UFSM 0329 and maintaining the same height in the larger specimens (Figure 6). The anterior tip of the snout seems to be equally quadrangular in all specimens with a preserved rostrum (Figure 4). The premaxilla is not preserved in CAPPA/UFSM 0191 (i.e., the smaller specimen), so we cannot confirm if this is the case for younger individuals. During ontogeny, the maxillary platform becomes more laterally developed (Figure 5). The interorbital space becomes wider in relation to the orbits, resulting in more separated eyes in large specimens (especially CAPPA/UFSM 0032 and CAPPA/UFSM 0330). The postorbital crests that border the interorbital depression of the skull roof become thicker and coarser, and the depression becomes slightly more pronounced. The orbits are large and rounded in the small CAPPA/UFSM 0191, growing slower than the skull and being proportionally smaller in large individuals (e.g., CAPPA/UFSM 0330, CAPPA/UFSM 0109) (Figure 4).

**Figure 7:**
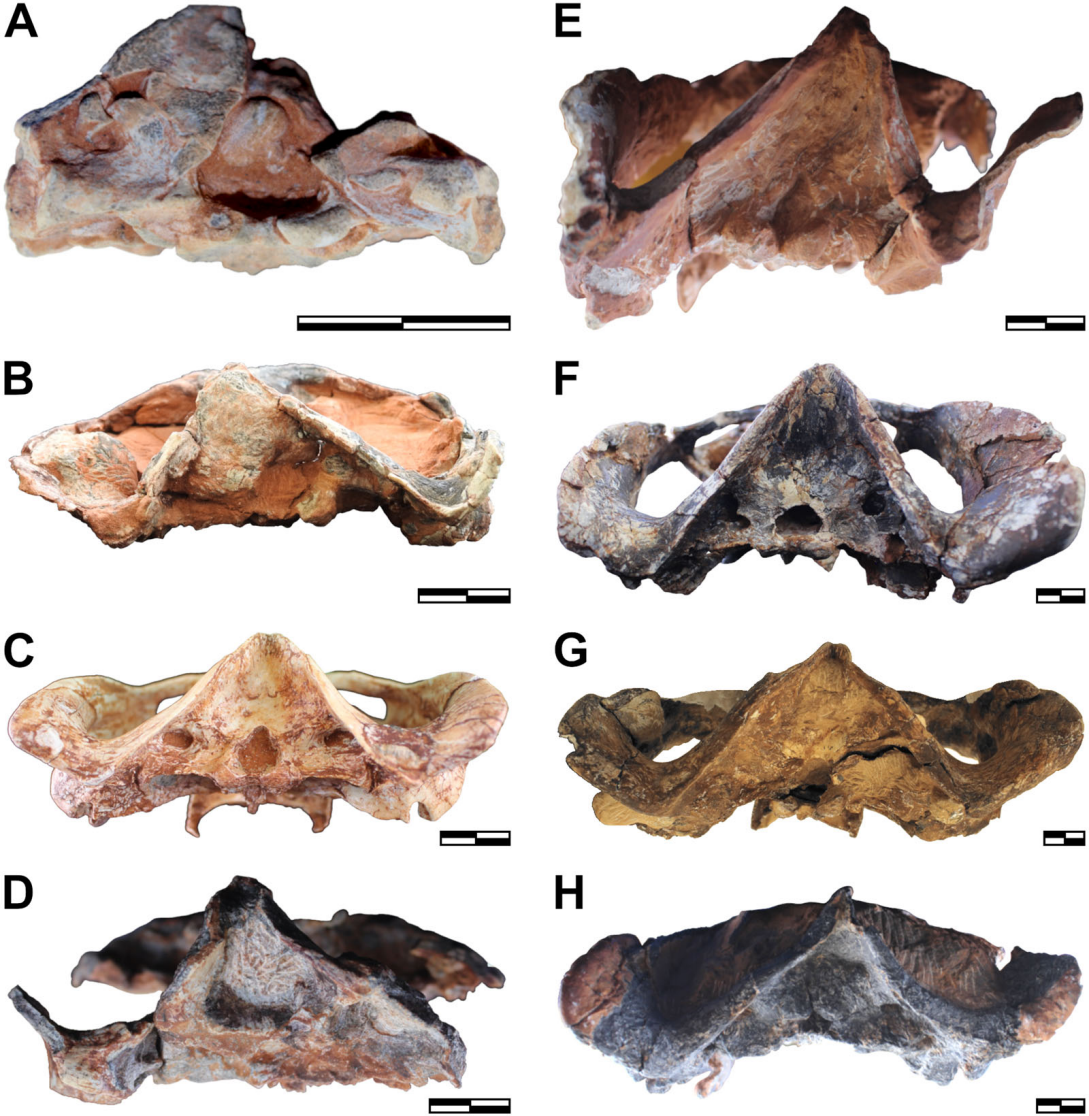
Crania of *Siriusgnathus niemeyerorum* in occipital view, in ascending order of size. (A) CAPPA/UFSM 0074; (B) CAPPA/UFSM 0191; (C) CAPPA/UFSM 0329; (D) CAPPA/UFSM 0394; (E) CAPPA/UFSM 0125; (F) CAPPA/UFSM 0032; (G) CAPPA/UFSM 0330; (H) CAPPA/UFSM 0109. Scale bars = 20 mm.

The zygomatic arches present many modifications throughout the growth of *S. niemeyerorum*. At first, they exhibit rounded anterior portions with a gradual departure from the rostrum, and a straighter and slightly divergent posterior portion (CAPPA/UFSM 0191) (Figures 4, 5 and 5). Intermediate individuals (CAPPA/UFSM 0329) lose this rounded anterior portion, departing abruptly from the rostrum. Their posterior portion remains somewhat straight and slightly divergent in dorsal view. In larger specimens (i.e., CAPPA/UFSM 0032, CAPPA/UFSM 0330, CAPPA/UFSM 0109), the simultaneous development of the suborbital region and the posteriormost portion of the squamosal results in a concave outline in dorsal and ventral views. In addition, the zygomatic arch becomes higher and more robust with ontogenetic growth (Figures 6, 7 and 7). Together, these changes give the cranium of the young *Siriusgnathus* an overall rounder appearance, which becomes more rectangular as the individual grows.

The ventral margin of the zygomatic arch is more horizontal in the small CAPPA/UFSM 0191, becoming more posteroventrally projected in larger specimens (Figure 6). The suborbital process of the jugal is absent in CAPPA/UFSM 0191. In CAPPA/UFSM 0329 (an intermediate specimen), there is a subtle ventral projection on the ventral margin of the jugal posterior to the suborbital region. The anterior margin of this projection is contiguous with the margin of the zygomatic arch, and its posterior margin retreats dorsally. In large specimens (i.e., CAPPA/UFSM 0032, CAPPA/UFSM 0330, CAPPA/UFSM 0260), this projection becomes more pronounced (but still rounded and gradual) and slightly more anteriorly located. It projects lateroventrally, becoming visible in dorsal view (Figure 4), which is not the case for CAPPA/UFSM 0329. In the largest specimen available (CAPPA/UFSM 0109), this is even more pronounced, but far from reaching the level of development of the suborbital process of *Exaeretodon riograndensis*.

The shape of the sagittal crest is relatively constant throughout ontogeny. It is slightly taller than the rest of the cranium profile, with its highest elevation at its posterior portion (Figure 6). The pineal foramen is absent in all specimens (Figure 4). The shape of the temporal fenestra changes throughout ontogeny (Figure 5). In the smaller specimen (CAPPA/UFSM 0191), it is rounded in ventral view, forming roughly an equilateral triangle. As the individual grows, the lateral face of this triangle increases faster (a consequence of the change in shape and growth of the zygomatic arch), giving the fenestra a more elongated aspect, as already seen in the intermediate specimen CAPPA/UFSM 0329, and bigger specimens (especially CAPPA/UFSM 0032 and CAPPA/UFSM 0330) (Figure 5).

**Figure 8:**
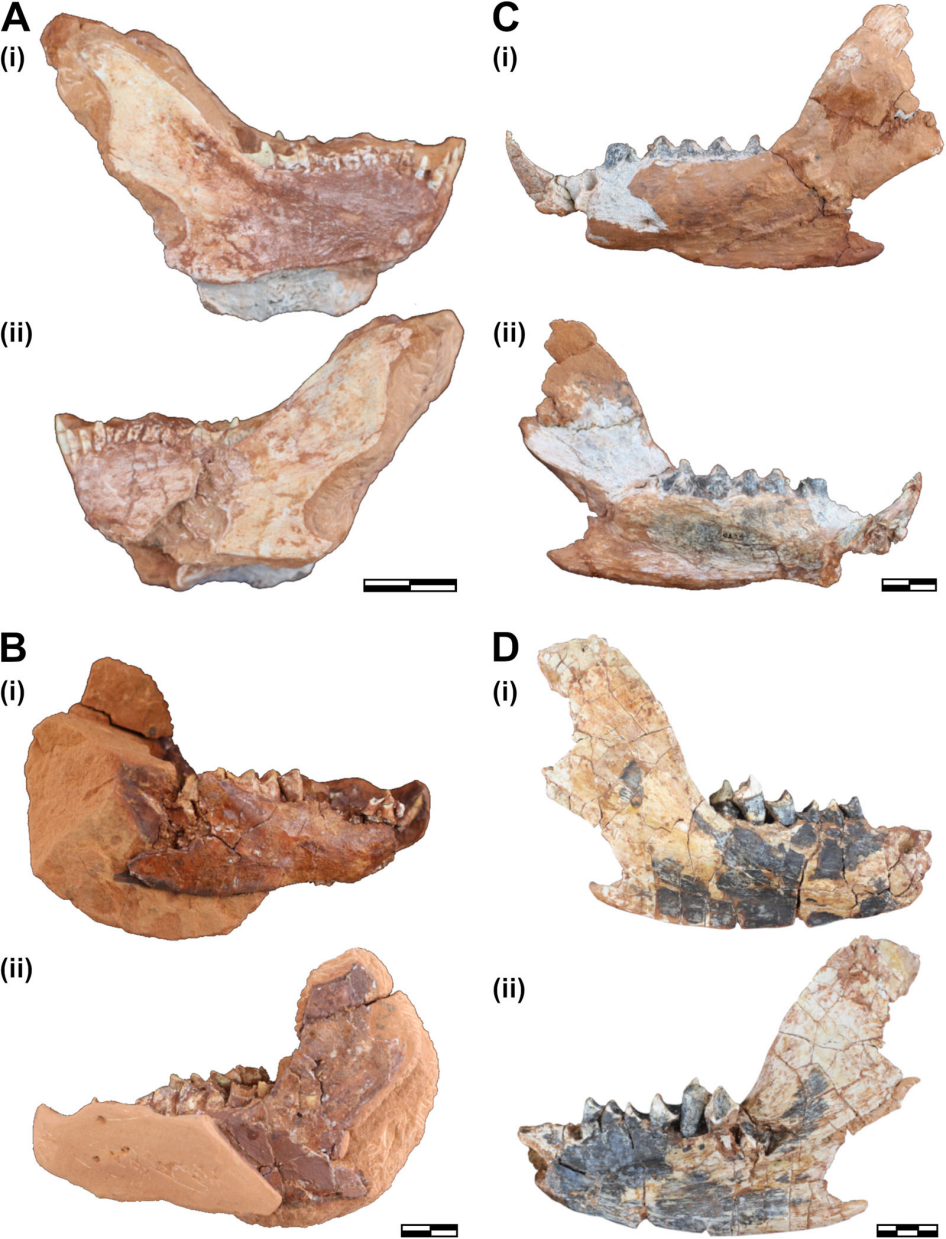
Lower jaw of *Siriusgnathus niemeyerorum*. (A) CAPPA/UFSM 0334 in lateral right (i), and lateral left (ii) views; (B) CAPPA/UFSM 0261 in lateral right (i) and lateral left (ii) views; (C) CAPPA/UFSM 0125 in lateral left (i) and medial left (ii) views; (D) CAPPA/UFSM 0032 in lateral right (i) and medial right (ii) views. Scale bars = 20mm (A–C) and 30mm (D).

The basioccipital and basisphenoid are fused even in the smaller specimens (i.e., CAPPA/UFSM 0074 and CAPPA/UFSM 0103), indicating that this fusion likely happens early in ontogeny (Figure 8). In contrast, the suture of the basioccipital + basisphenoid complex with the prootic and the opisthotic is markedly open in these specimens. At this stage, another conspicuous open suture separates the basioccipital and the exoccipital (occipital condyles). In the intermediate specimen (CAPPA/UFSM 0329), these sutures are already closed but still visible. In CAPPA/UFSM 0032 (i.e., the larger specimen with a well‐preserved basicranium), the suture between the occipital condyles and the basioccipital is blurred, indicating the advanced degree of fusion between these bones.

In the occiput, the base of the lambdoidal crest becomes more ventrally and anteriorly deep, partially a result of the posterior development of the zygomatic process of the squamosal (Figures 4, 7 and 7). The shape of the foramen magnum varies amongst specimens, but this variation does not follow a clear ontogenetic trajectory. In the small CAPPA/UFSM 0074 (presumably juvenile) and CAPPA/UFSM 0330 (one of the larger specimens), it is roughly triangular, with a mediolaterally enlarged oval base and a narrower but still rounded dorsal portion. In CAPPA/UFSM 0329 and CAPPA/UFSM 0125 (medium‐sized specimens), this dorsal rounded portion is narrower, and the triangular shape is more accentuated. Contrastingly, in the large CAPPA/UFSM 0032, the foramen magnum is completely oval. However, the relative size of the foramen follows a clearer ontogenetic trend, occupying a larger portion of the occipital plate in young specimens in relation to older ones (i.e., it increases in size slower than the cranium).

**Figure 9:**
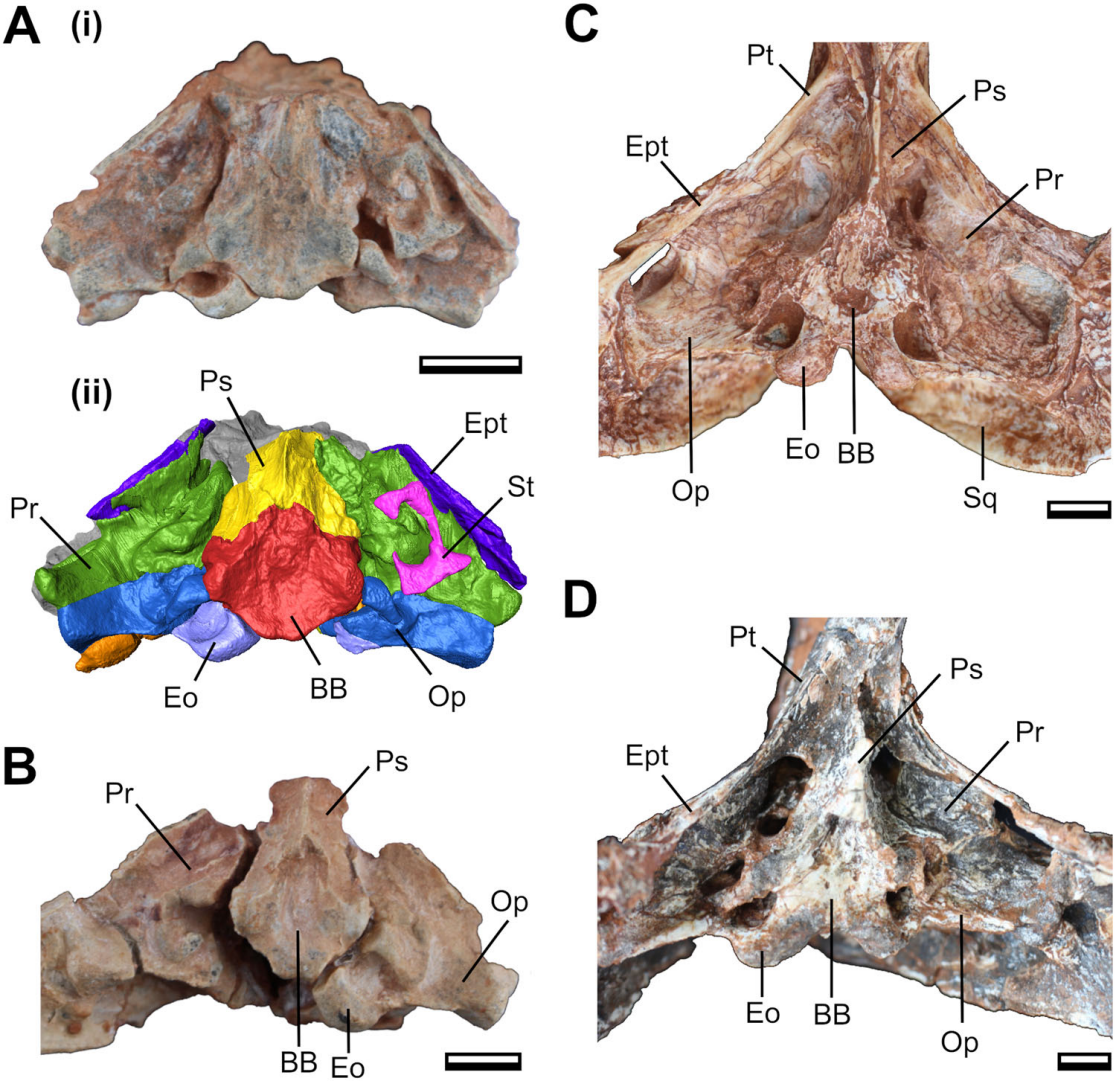
Basicranial anatomy of *Siriusgnathus niemeyerorum* in ventral view. (A) CAPPA/UFSM 0074: photograph (i) and 3D digital models (ii); (B) CAPPA/UFSM 0103; (C) CAPPA/UFSM 0329; (D) CAPPA/UFSM 0032. Scale bars = 10 mm. BB, basisphenoid + basioccipital; Eo, exoccipital; Ept, epipterygoid; Op, opisthotic; Pr, prootic; Ps, parasphenoid; Pt, pterygoid; Sq, squamosal; St, stapes.

The comparison of the lower jaw of the small CAPPA/UFSM 0334 with larger specimens (CAPPA/UFSM 0125 and CAPPA/UFSM 0032) highlights a few patterns in mandibular anatomy (Figure 9). The masseteric fossa is equally shallow in both groups. The inclination of the coronoid process is less pronounced in CAPPA/UFSM 0334, and the process does not become as high as in the large specimens. The angular process is also less posteriorly developed in CAPPA/UFSM 0334. The lower incisors are small and only slightly larger than the canines in CAPPA/UFSM 0334, while they are larger and more procumbent in CAPPA/UFSM 0125. Although there is a significant size difference, both the small (presumably juvenile) CAPPA/UFSM 0334 and the large (presumably adult) CAPPA/UFSM 0032 have the same number of lower postcanines (7 + 1 in eruption). Similarly, all cranial specimens with the preserved tooth row exhibit the same number of upper postcanines (8 + 1 in eruption) (Figure 5), highlighting a considerable ontogenetic conservativism in postcanine number in *S. niemeyerorum*.

## 4 Discussion

**Figure 10:**
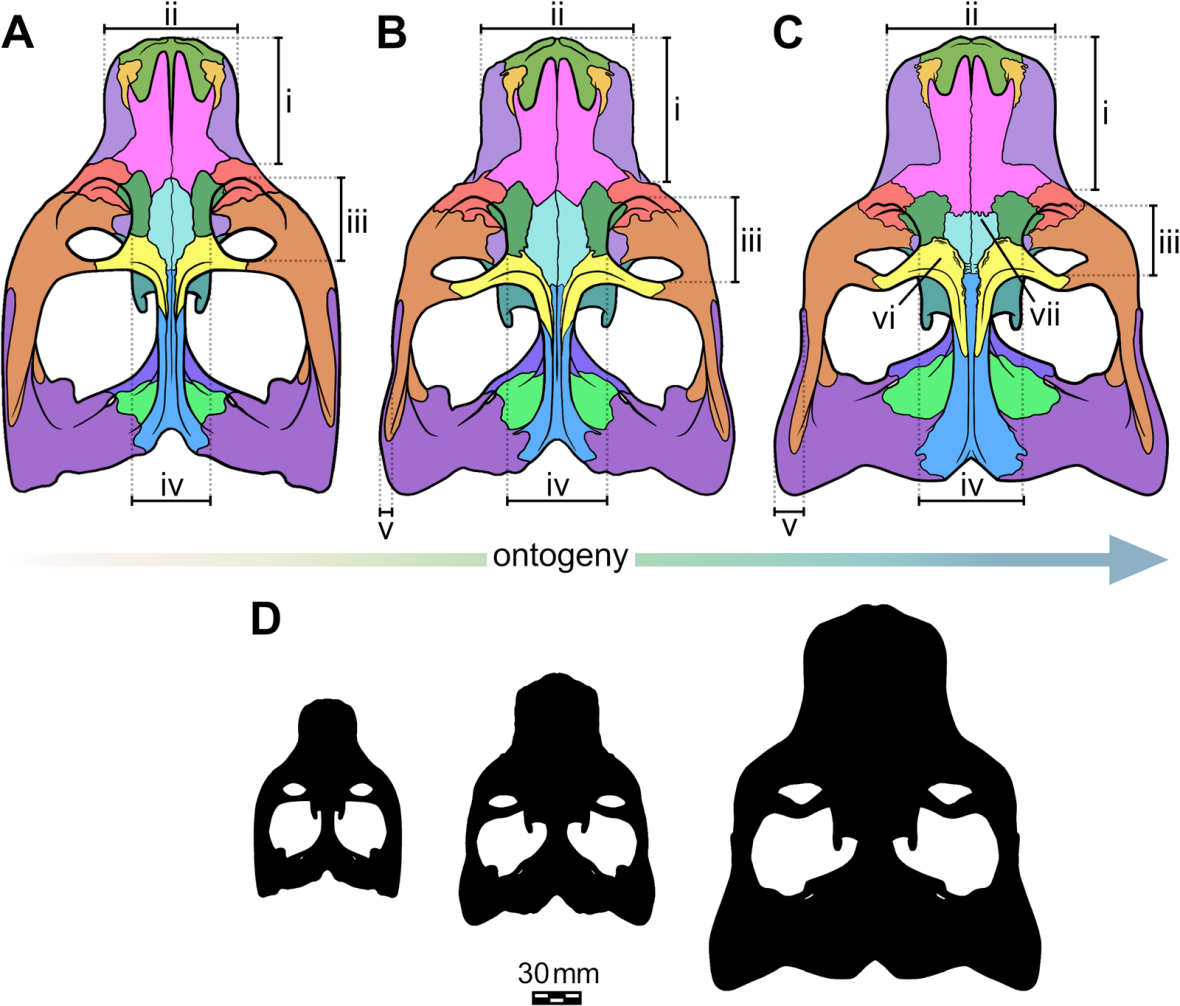
Hypothetical reconstruction of the ontogenetic trajectory of the cranium of *Siriusgnathus niemeyerorum*. (A) juvenile, anatomy based on CAPPA/UFSM 0191 and 0124. (B) young adult, anatomy based CAPPA/UFSM 0329 (sutures based on Roese‐Miron et al. in review). (C) old adult, anatomy based on CAPPA/UFSM 0032, 0109 and 0330. (A–C) are not to scale. (D) outlines to scale of the three hypothetical specimens depicted in (A–C) based on the size of CAPPA/UFSM 0191, CAPPA/UFSM 0329 and CAPPA/UFSM 0330, respectively. i, elongation of the rostrum; ii, increase in rostrum width; iii, decrease in orbit relative size; iv, increase in interorbital width; v, lateral development of the posterior portion of the zygomatic arches; vi, increase in cranial ornamentation; vii, increase in suture complexity.

Our results suggest a few patterns in skull growth during the ontogeny of *S. niemeyerorum* (see Figure 10). The skull width increases approximately isometrically with the skull length, the same pattern as in *Exaeretodon argentinus* (Wynd et al. 2022). Additionally, we observe that as the skull increases: (i) the rostrum becomes proportionally longer and wider; (ii) the orbit becomes proportionally smaller; (iii) the zygomatic arch length grows slightly slower than skull length, but its height increases faster; (iv) the posterior portion of the zygomatic arch develops laterally, increasing the width of this region faster than the orbital region; (v) the temporal region elongates relatively isometrically with skull length; (vi) the temporal fenestra becomes larger both in length and width in proportion to skull length; (vii) the secondary palate and the PC row become proportionally shorter; (viii) the distal PC distance and transverse process width become proportionally narrower; and (ix) the basicranial region becomes proportionally smaller.

Here, we observed that the smaller specimens have proportionally larger orbits than the adults. Juveniles with larger eyes than their adult counterparts are a widespread pattern in vertebrates (e.g., Fernandez Blanco et al. 2018; Yuan et al. 2021; Hone and McDavid 2025), and it has been proposed as the plesiomorphic state for Tetrapoda (Fernandez Blanco et al. 2018). Accordingly, this has been reported for many traversodontids (Liu 2007; Abdala and Giannini 2000; Liu et al. 2008; Kammerer et al. 2012). This pattern could be explained by the functional role of the eye, which must be a certain size to allow enough light to enter to enable sight (Walls 1942; Griffin et al. 2021).

The proportional increase in rostrum length during ontogeny is also common amongst vertebrates (see Griffin et al. 2021), and it has already been reported for some other cynognathians (Brink 1963; Sues and Hopson 2010; Kammerer et al. 2012). However, this feature is not as ubiquitous, and even some cynodonts exhibit a pattern closer to isometry (or even negative allometry), such as *Massetognathus* spp., *Menadon besairiei*, and *Exaeretodon riograndensis* (Abdala and Giannini 2000; Kammerer et al. 2008; Liu et al. 2008; Wynd et al. 2022).

The temporal and zygomatic regions are attachment points for the adductor musculature in the cranium, and thus have a direct impact on how we interpret these soft tissues. Wynd et al. (2022) correlated the temporal length with the development of the m. temporalis (which attaches to the cranium on the parietal crest), and the temporal width and zygoma height with the m. masseter (which attaches to the cranium on the medial surface of the zygomatic arch) (Lautenschlager et al. 2017). For instance, a faster increase in the mass of the m. temporalis has been proposed for *Massetognathus* spp., given the positive allometry of its temporal length and the development of the parietal crest (Abdala and Giannini 2000; Liu et al. 2008).

A different pattern is seen in *S. niemeyerorum*, in which the length of the temporal region grows almost isometrically. In the species, the length of the zygomatic arch grows slightly slower than the skull length, while the arch becomes higher and more laterally developed (with the latter also occurring in *Menadon besairiei* and *E. argentinus;* Kammerer et al. 2008; Wynd et al. 2022). At the same time, the temporal fenestra becomes longer and wider, like in *Massetognathus ochagaviae*, *Dadadon isaloi*, and *Exaeretodon argentinus* (Liu et al. 2008; Kammerer et al. 2012; Wynd et al. 2022). The increase in temporal width, coupled with the increase in the height and lateral development of the zygomatic arch indicates that the m. masseter is likely developing at a faster pace in *S. niemeyerorum*.

As has been proposed for *E. argentinus* (Wynd et al. 2022), this may indicate a dietary shift from more insectivorous juveniles (with a crushing feeding style associated with the development of the m. temporalis) to more herbivorous adults (with a chewing feeding style, associated with the m. masseter). The possibility of such a shift has also been raised for other cynognathians such as *Diademodon tetragonus* (Brink 1955; Grine et al. 1978) and *Cricodon metabolus* (Sidor and Hopson 2017). The opposite pattern is found in the epicynodont *Thrinaxodon liorhinus* and in *Massetognathus* spp. (Liu et al. 2008; Jasinoski et al. 2015): the zygomatic height increases slower than the temporal length, indicating that the m. temporalis becomes more prominent than the m. masseter. This indicates that different traversodontid lineages may have adopted different feeding trajectories while growing, which could be related to their different absolute size and/or growth strategies (Chinsamy and Abdala 2008; Veiga et al. 2018; Garcia Marsà et al. 2022).

After analysing a more encompassing series of *S. niemeyerorum* specimens, we believe that the presence of the suborbital process in the species—so far considered present but little projected (Pavanatto et al. 2018, #13; Hendrickx et al. 2020, #133)—is debatable. The lateral outline of the zygomatic arch of the smaller specimen (i.e., CAPPA/UFSM 0191) is similar to that of *Massetognathus* spp., in which the suborbital process is considered absent (Romer 1967; Liu et al. 2008) (Figures 4, 6 and 6). In CAPPA/UFSM 0329, there is a ventral development on the jugal that could be interpreted as an inconspicuous suborbital process, but two factors hinder this attribution: first, it is more posteriorly oriented than the suborbital process of bigger specimens; second, its anterior margin is continuous with the margin of the zygomatic arch, instead of forming a proper salient process. It is only in the larger specimens, such as the holotype and CAPPA/UFSM 0109, that a proper (yet underdeveloped) process is formed. It protrudes gradually from the zygomatic arch, but it does become differentiated from the arch and noticeable in dorsal view. Given that the suborbital process is the point of insertion for the m. masseter pars superficialis, this is another evidence supporting the ontogenetic development of the masseteric muscles in *S. niemeyerorum* (see also Abdala and Damiani 2004).

The development of the suborbital process of the jugal has been used as a phylogenetic character for traversodontids, despite being known to be variable within some species (e.g., *Massetognathus pascuali*, Schmitt et al. 2019; *Exaeretodon* spp., Schmitt et al. 2023). However, this intraspecific variation is usually a matter of shape and degree of development, but the presence (or absence) of the feature seems to be consistent within a species (Melo et al. 2022; Schmitt et al. 2023; Kerber et al. 2024). This distinction may be more evident in species with the ‘extreme’ states: absent (e.g., *Massetognathus ochagaviae*) (Barberena 1981; Liu et al. 2008), or conspicuously projected/ball‐like (e.g., *E. riograndensis*, *Santacruzodon hopsoni*) (Abdala et al. 2002; Abdala and Ribeiro 2003). However, for species such as *S. niemeyerorum* in which the character is intermediate (i.e., present but little projected), the characterization can become more subjective. Our results show that the scoring of this character could vary based on the ontogenetic stage of the specimen. Evidence of the development of the suborbital process during ontogeny has also been seen in the early‐divergent cynodont *Galesaurus planiceps* and the trirachodontid *Trirachodon berryi* (Abdala and Damiani 2004). Furthermore, an analysis of other traversodontids exhibiting this character state (e.g., *Andescynodon mendozensis* and *Pascualgnathus polanskii*), as well as the basal cynognathian *Cynognathus crateronotus*, reveals that the distinction between ‘slightly projected’ and ‘conspicuously projected’ is dubious, particularly when considering intraspecific variation. Therefore, we emphasize the need for a comprehensive re‐evaluation of this character in traversodontids.

Regarding the palatal region, the secondary palate length, PC row length, and transverse process width grow slower than the total skull in *S. niemeyerorum*. Wynd et al. (2022) note that in *E. argentinus*, in which a similar pattern is observed, the reduction in the transverse process width indicates a narrower positioning of the lower jaw in the skull, which would be related to a more developed occlusal musculature. The distance between the more distal PCs also decreases with age in *S. niemeyerorum*, similar to *M. pascuali* (Abdala and Giannini 2000).

The changes in basicranium size (slower growth than overall cranium) could reflect a possible tendency of slower growth in the brain in relation to the skull. A decrease in the relative size of the brain throughout ontogeny is a common trend across vertebrates (e.g., Hurlburt et al. 2013; Lautenschlager and Hübner 2013; Jirák and Janaček 2017; Watanabe et al. 2019; Dumont et al. 2020; Hu et al. 2021; Ferreira et al. 2022). Thus, it would not be surprising if that is the case for *S. niemeyerorum*, although this hypothesis would have to be investigated directly.

The degree of cranial ornamentation, plus the fusion and complexity of the cranial sutures, are also commonly associated with age (e.g., Chatterjee 1982; Jasinoski et al. 2015. Jasinoski and Abdala 2017). We found that the frontal‐postorbital crests develop later in life in *S. niemeyerorum*, with medium‐sized specimens (i.e., CAPPA/UFSM 0329) still exhibiting poorly developed crests, and, consequently, a shallow interorbital depression. A very similar pattern was also recorded for *Dadadon isaloi* (Kammerer et al. 2012). As an individual grows, the sutures between the bones tend to become more strongly fused, and many become indistinguishable. Furthermore, they can also become highly interdigitated, which increases the attachment of the bones and, consequently, can help with skull integrity while it endures stresses resulting from mastication forces (Lautenschlager et al. 2018; Wynd et al. 2022).

We noted that many sutures already exhibit an intricate interdigitated pattern in CAPPA/UFSM 0329, such as the prootic‐epipterygoid and the lacrimal‐jugal sutures. The fusion appears to increase even more in the larger specimens, given that the aforementioned sutures, as well as the sutures in the basicranium, palate and occiput, become practically indistinguishable. It is unlikely that this is purely a preservation bias, because the sutures in the skull roof are still visible in the larger specimens. These sutures are relatively simple in CAPPA/UFSM 0329, which is partially maintained in the larger specimens (except for the frontal‐nasal suture, which becomes more complex).

Our results also provide a general framework for the sequence of suture closure in the basicranium of *S. niemeyerorum*. The basisphenoid and basioccipital fuse completely early in ontogeny. The prootic‐epipterygoid and the prootic‐opisthotic sutures also close early, but they remain visible until further in ontogeny. The only still completely open sutures in the presumed juveniles (CAPPA/UFSM 0074 and CAPPA/UFSM 0103) are of the basioccipital + basisphenoid with the exoccipital and the prootic, showing that they close later in life.

As previously noted by Pavanatto et al. (2018), the number of upper postcanines is notably consistent during the ontogeny of *S. niemeyerorum*, with all specimens with a preserved tooth row presenting 9 PCs (8 + 1 in eruption). Some degree of ontogenetic variation in the number of postcanines is expected in traversodontids, with species such as *Boreogomphodon jeffersoni, Dadadon isaloi*, *Massetognathus pascuali*, and *Santacruzodon hopsoni* increasing the number (Abdala and Giannini 2000; Sues and Hopson 2010; Kammerer et al. 2012; Melo et al. 2022), and *Exaeretodon argentinus*, *Exaeretodon riograndensis* (to a lesser extent), and possibly *Menadon besairiei* decreasing (Kammerer et al. 2008; Melo et al. 2015; but see Flynn et al. 2000). It is noteworthy that the smaller specimen in our sample (i.e., CAPPA/UFSM 0191) does not preserve the upper postcanines alveoli, so we cannot determine if there is a change in the number of PCs at the earlier stages of ontogeny.

It is important to highlight that although the changes observed here most likely reflect general ontogenetic trends, there are caveats about interpreting all variation as developmental in nature. For example, factors such as individual variation and sexual dimorphism can affect the spectre of variation of a species, which imposes an even greater challenge for fossil samples (Grine et al. 1978; Jasinoski and Abdala 2017). Nevertheless, the patterns found here are mostly consistent with the ontogeny of a range of non‐mammalian cynodonts, which increases their support.

## 5 Conclusions

**Figure 11:**
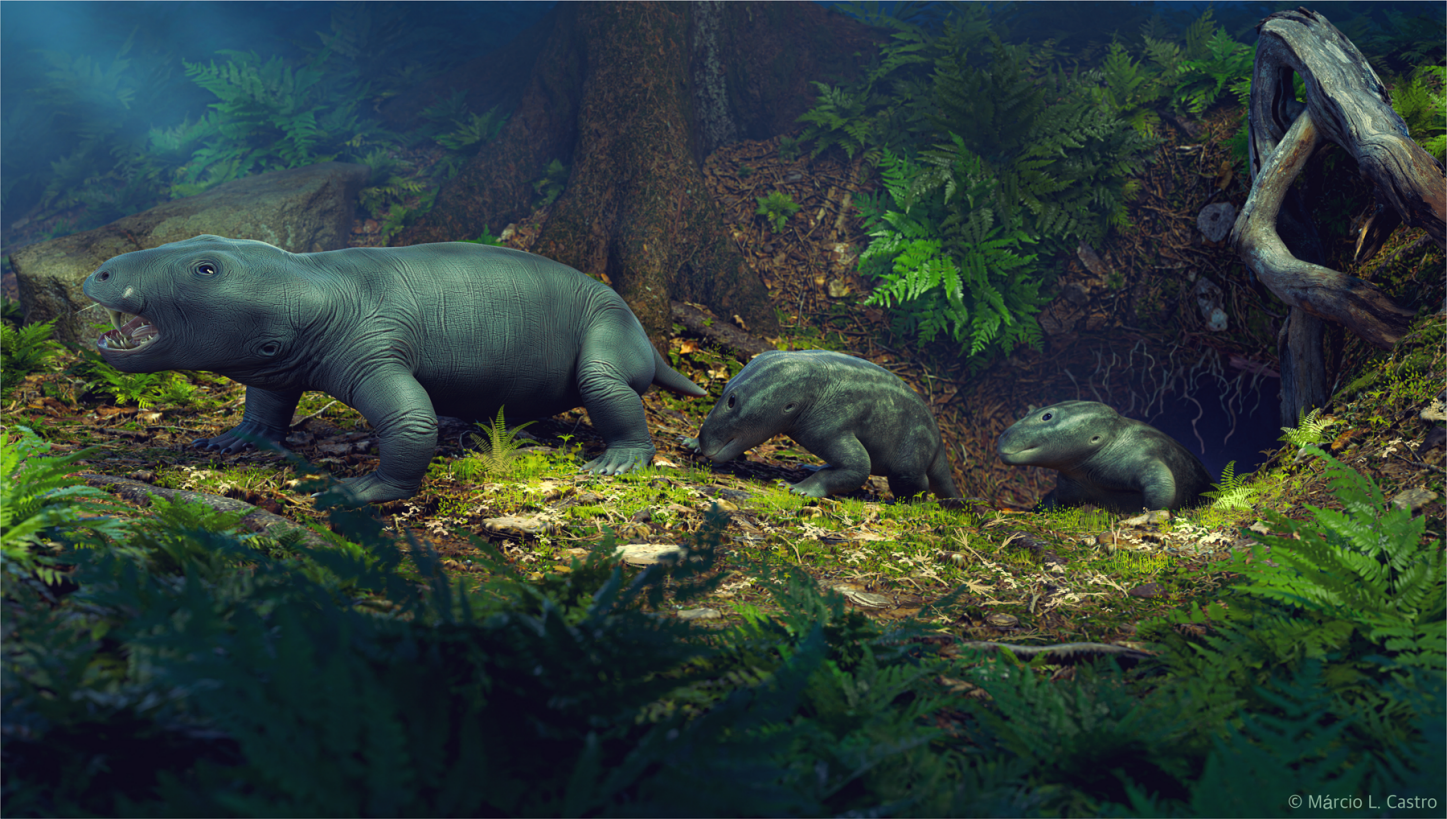
Artistic representation of three individuals of *Siriusgnathus niemeyerorum* in a Late Triassic landscape of southern Brazil. The adult (on the left) is followed by two juveniles. Artwork by Márcio L. Castro.

In this study, we described ontogenetic trends in the shape and anatomy of the skull of the Late Triassic South American traversodontid *S. niemeyerorum* (Figure 11) and suggested possible functional and paleoecological implications based on our findings. 

We identified several allometric and morphological trends that take place as the individual ages. These include a change in general skull shape, from rounded, more gracile crania in juveniles to quadrangular, more ornamented ones in adults. It also encompasses some patterns that are shared with other cynognathians, such as the increase in rostrum length, temporal fenestra size, and zygomatic arch height, and the decrease in orbital dimensions. By correlating these morphological features with muscle attachment sites, our findings potentially indicate a change in muscle usage—and thus, mastication patterns—during growth.

## Author Contributions

**Lívia Roese‐Miron:** conceptualization, investigation, writing – original draft, methodology, visualization, writing – review and editing, formal analysis. **Leonardo Kerber:** conceptualization, writing – review and editing, supervision, resources.

## Conflicts of Interest

The authors declare no conflicts of interest.

## Data Availability

All of the original contributions from this study are available in the body of the work. Further inquiries may be requested to the corresponding author.
